# Assessing Random Forest self-reproducibility for optimal short biomarker signature discovery

**DOI:** 10.1093/bib/bbaf318

**Published:** 2025-07-11

**Authors:** Ahmed Debit, Christophe Poulet, Claire Josse, Guy Jerusalem, Chloe-Agathe Azencott, Vincent Bours, Kristel Van Steen

**Affiliations:** Laboratory of Human Genetics, GIGA Institute, University of Liege (ULiege), Avenue Hippocrate 1/11, 4000 Liege, Belgium; BIO3, GIGA Institute, University of Liege (ULiege), Avenue Hippocrate 1/11, 4000 Liege, Belgium; Institut de Biologie de l’ENS (IBENS), Ecole Normale Superieure, 46 rue d'Ulm, 75005 Paris, France; Department of Rheumatology, GIGA Institute, University Hospital of Liege (CHULiege), University of Liege (ULiege), Avenue Hippocrate 1/11, 4000 Liege, Belgium; Fibropole Research Group, University Hospital of Liege (CHULiege), Avenue de l'Hopital 1, 4000 Liege, Belgium; GIGA-I3 Research Group, GIGA Institute, University of Liege (ULiege) and University Hospital of Liege (CHULiege), Avenue Hippocrate 1/11, 4000 Liege, Belgium; Laboratory of Human Genetics, GIGA Institute, University of Liege (ULiege), Avenue Hippocrate 1/11, 4000 Liege, Belgium; Oncology Department, University Hospital of Liege (CHULiege), University of Liege (ULiege), Avenue de l'Hopital 1, 4000 Liege, Belgium; Oncology Department, University Hospital of Liege (CHULiege), University of Liege (ULiege), Avenue de l'Hopital 1, 4000 Liege, Belgium; Mines Paris, PSL Research University, CBIO-Centre for Computational Biology, 60 boulevard Saint-Michel, F-75006 Paris, France; Institut Curie, PSL Research University, 26 rue d'Ulm, F-75005 Paris, France; Inserm, U900, 26 rue d'Ulm, F-75005 Paris, France; Laboratory of Human Genetics, GIGA Institute, University of Liege (ULiege), Avenue Hippocrate 1/11, 4000 Liege, Belgium; Center for Human Genetics, University Hospital of Liege (CHULiege), Avenue de l'Hopital 1, 4000 Liege, Belgium; BIO3, GIGA Institute, University of Liege (ULiege), Avenue Hippocrate 1/11, 4000 Liege, Belgium

**Keywords:** cancer diagnosis signature, biomarker, short signature discovery, AUC stability, Random Forest reproducibility

## Abstract

Biomarker signature discovery remains the main path to developing clinical diagnostic tools when the biological knowledge on pathology is weak. Shortest signatures are often preferred to reduce the cost of the diagnostic. The ability to find the best and shortest signature relies on the robustness of the models that can be built on such a set of molecules. The classification algorithm that will be used is often selected based on the average Area Under the Curve (AUC) performance of its models. However, it is not guaranteed that an algorithm with a large AUC distribution will keep a stable performance when facing data. Here, we propose two AUC-derived hyper-stability scores, the Hyper-stability Resampling Sensitive (HRS) and the Hyper-stability Signature Sensitive (HSS), as complementary metrics to the average AUC that should bring confidence in the choice for the best classification algorithm. To emphasize the importance of these scores, we compared 15 different Random Forest implementations. Our findings show that the Random Forest implementation should be chosen according to the data at hand and the classification question being evaluated. No Random Forest implementation can be used universally for any classification and on any dataset. Each of them should be tested for their average AUC performance and AUC-derived stability, prior to analysis.

## Graphical Abstract

**Graphical Abstract:**
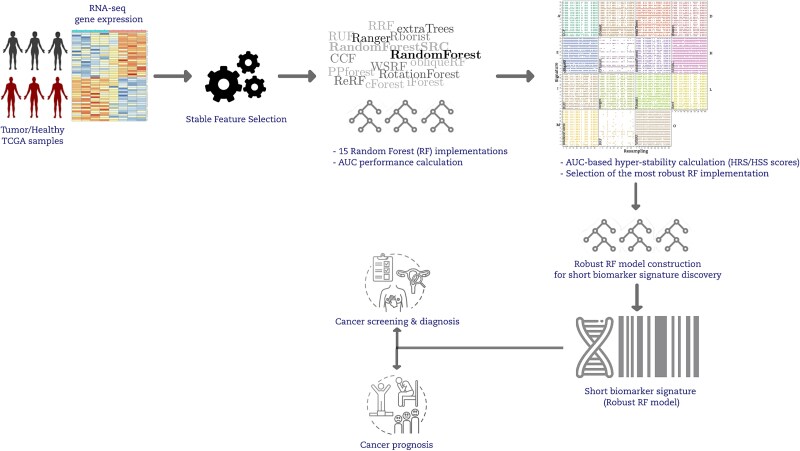

## Introduction

In the field of cancer care, biomarker screening helps clinicians in their decision-making. Biomarker Signature Discovery (BSD) aims to identify a set of hundreds of variables, out of thousands, that will capture the molecular differences between the categories of patients to study. Short BSD focuses on the most relevant variables to build the shortest predictive signature for comfortable use in daily clinical routine. While the clinicians expect a manageable test with few variables, they also expect it to be robust enough to reduce the prediction error.

Short combinations of biomarkers, also called short signatures, can be easily transferred to daily clinical routines. These short signatures can be used at a low cost to diagnose cancer subtypes, predict treatment responses, or monitor patients during treatment [1, 2]. However, with >10 000 clinical trials, based on biomarkers and cancer, currently ongoing [3], only a few studies may be successfully transferred to the clinics, and fewer may impact the diagnosis practices as only a few biomarkers are clinically relevant yet [4]. Indeed, within the past clinical trials on Breast Cancer, only the Oncotype DX, MammaPrint, EndoPredict, Breast Cancer Index (BCI), and Prosigna (PAM50) multianalyte tests have been successfully transferred with their associated model of prediction [5]. Despite these few commercial successes, many publications are directly related to biomarkers [4], and the design of short, robust, and universal signatures of biomarkers predictive of a clinical state remains challenging. Combinatory strategies are increasingly used to determine multivariate signatures [1, 6]. Machine Learning (ML) has gained popularity in this sense to create associated models and assess the robustness of those signatures [6, 7]. Researchers often compare ML strategies upstream to determine the “best” approach to use and usually rely on the highest average AUC performance. However, such average AUCs are highly variable. Indeed, based on Gonzalez-Bosquet *et al*.’s observation, nine ML methods, including RF, exhibited AUCs ranging from 0.53 to 0.73 [8]. Due to this variation, the average AUC is not an optimal criterion for assessing the best ML methodology.

Random Forest (RF) is among the most popular machine learning methods in bioinformatics and related fields. RF is an ensemble of classification or regression trees that was introduced by Breiman [9]. It is extensively applied to gene expression data because it copes with ‘large p small n’ problems, it exhibits relatively good accuracy, is robust to noise, and requires little parameter tuning. Moreover, RF is easy to use and the interpretation of the resulting models is facilitated since it is all about a suite of ‘if .. else’ -like decision rules. Since the original RF algorithm proposed by Breiman [9], several variations to RFs have been made available via the R Project for Statistical Computing, including orthogonal and oblique methods.

The current study aimed at assessing RF strategies based on both the average AUC and stability of the resulting AUC. Therefore, the question asked was how can AUC stability help in deciding the best predictive RF implementation? We put this question in the context of a short BSD identification and evaluation. In the BSD field, several applications of RFs exist [6, 8]. In this study, we focus on assessing the most stable RF method, from 15 implementations in R (Table 1). Our study was driven by tumor versus healthy in paired samples from The Cancer Genome Atlas (TCGA) database (RNAseq of Breast Cancer (BRCA), Lung Squamous cell Carcinoma (LUSC), and Thyroid Cancer (THCA) cancers).

**Table 1:** Summary of RF methods implemented in R language and used in this study.

**Implementation**	**Algorithm**	**OOB**	**R package**
RandomForest	Orthogonal	✔	randomForest v4.6-12 [10]
RFSRC	Orthogonal	✔	randomForestSRC v2.6.1 [11]
Ranger	Orthogonal	✔	ranger v0.10.1 [12]
cForest	Orthogonal	✔	partykit v1.1.1 [13]
Rborist	Orthogonal	✔	Rborist v0.1-8 [14]
ExtraTrees	Orthogonal		extraTrees v1.0.5 [15]
RUF	Orthogonal	✔	randomUniformForest v1.1.5 [16]
RRF	Orthogonal	✔	RRF v1.7 [17]
WSRF	Orthogonal	✔	wsrf v1.7.17 [18]
iForest	Orthogonal		iRF v.2.0.0 [19]
CCF	Oblique		ccf v1.0.0 [20]
PPForest	Oblique	✔	PPforest v0.1.1 [21]
ObliqueRF	Oblique	✔	obliqueRF v0.3 [22]
RotationForest	Oblique		rotationForest v0.1.3 [23]
Rerf	Oblique	✔	rerf v1.0 [24]

OOB, out-of-bag; RFSRC, RandomForestSRC; RUF, RandomUniformForest; RRF, Regularized Random Forest; WSRF, Weighted Subspace Random Forest; CCF, Canonical Correlation Forest; PPForest, Pursuit Projection Random Forest; Rerf, Randomer Forest.

The conclusions are two-fold. First, AUC-derived stability reveals the dataset dependency of an RF implementation. Second, based on two distinct scores, hyper-stability can highlight whether an RF implementation is signature or resampling dependent. Consequently, AUC stability provides a confidence score on top of the commonly used average AUC for the selection of the best RF implementation. Additionally, the modelization time can further help discriminating between RF implementations with equal stability performance.

## Materials and methods

The description of the symbols used in the following formulas is given in Table S1 in the supplementary data File S1.

### The Cancer Genome Atlas sample collection, normalization, and filtering

TCGA database was screened to maximize the number of paired tumor–healthy samples in cancer cohorts. TCGA clinical data were filtered to select cancers with the most similar histological subtypes and patients with paired healthy tumor samples. Subsequently, three TCGA datasets were used in this study: BRCA, LUSC, and THCA. These three datasets were downloaded using the TCGA2STAT R-package [25]. Paired healthy tumor samples were collected with Reads Per Kilobase per Million mapped reads (RPKM) normalization using the *tumorNormalMatch* function from the same R-package. The total number of primary variables was reduced based on their variance to ${\mathrm{N}}_{\mathrm{v}}$ variables before the feature selection step, using the Log Intensity variation function of the BRB-ArrayTools software (version 3.8.1) with the *P*-value parameter set to .001. The description of the three datasets selected was detailed in Table 2.

**Table 2:** Description of TCGA datasets used in this study. The result summary of Step 1 and Step 2 on each dataset is also reported.

**Dataset**	**TCGA-BRCA**	**TCGA-LUSC**	**TCGA-THCA**
**Dataset description**			
Histological subtype	Infiltrating ductal carcinoma	Lung squamous cell carcinoma	Thyroid papillary carcinoma
Patients	91	48	49
Samples	182	96	98
Number of variables ${N}_v$	9500	9262	9353
**Result of Step 1 and Step 2 of the pipeline**			
Number of variables after FS ${N}_{v\prime }$	28	9	38
Number of trees after OOBerr ${N}_t$	500	500	500
Selected signatures	78	21	108
Overall variable correlation	0.61	0.83	0.64
Highly correlated variables within signature (%)	0.02	0.96	0.03
Variable-to-sample ratio	0.3	0.2	0.8

### Workflow of the comparative study

**Figure 1:**
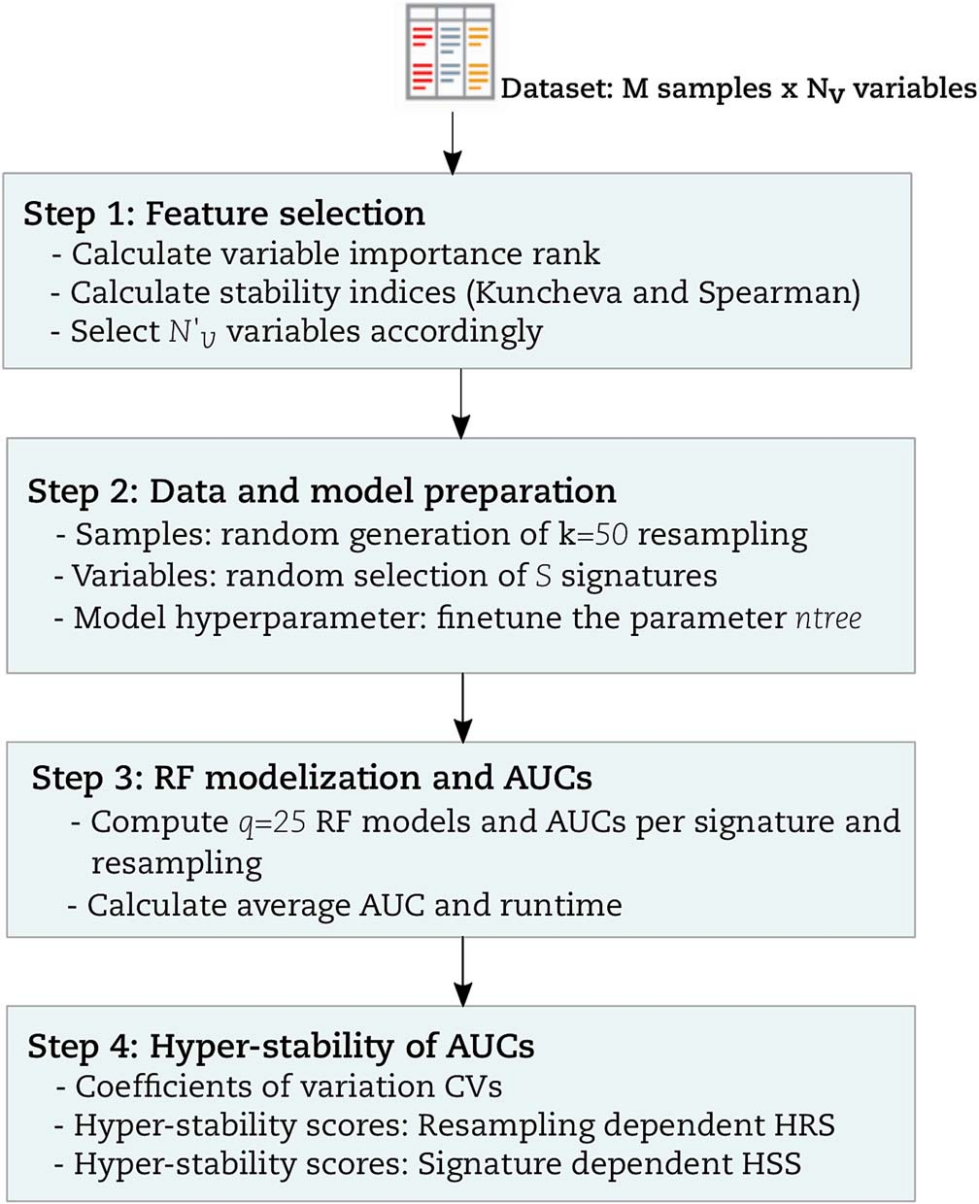
Overall procedure of AUC-based hyper-stability benchmarking of RF implementations.

A graphical summary of our study comparing multiple RF implementations via hyper-stability assessment is given in (Fig. 1). Next, we explain each step in greater detail. We use the term ‘resampling rate’ to refer to the percentage of data that goes to the training partition after a balanced random sampling without a replacement from the original data.

#### Step 1: Feature selection

The Feature Selection (FS) procedure used in this study followed the rank-based overlap and correlation principles inspired by Alelyani *et al*. [26]. FS was used to determine the minimum number of variables to be retained for downstream analysis. Rank-based variable importance was used and calculated based on a combination of the Mean Decrease in GINI (MDG) and Mean Decrease in Accuracy (MDA) of the RF algorithm. The threshold used for the selection of was determined using stability indices Kuncheva and Spearman. The entire methodology concerning FS is provided in the supplementary file (File S1).

#### Step 2: Data and model preparation

- *Fine-tune the RF parameter ntree*: For all RF methods that implement the out-of-bag principle (Table 1), the out-of-bag error (OOBerr) was computed based on variables as a function of *ntree* ∈ {10, 20, .., 1200}. Similarly, a total of *k* = 50 balanced random case/control partitions were generated using a resampling rate of *P = .9*, and *q* = 25 intrinsic RF models per partition. Subsequently, 50 × 25 = 1250 RF models and 1250 OOBerr values were obtained for each value of *ntree*. The *OOBerr* was averaged over the *ntree* value and plotted as a function of *ntree* for each RF method. The minimal number of trees ${N}_{\mathrm{t}}$ = 500 was obtained when the *OOBerr* was optimized and stabilized for the tested implementations. This value of is sufficiently acceptable from all the RF implementations including those not implementing the out-of-bag principle.
- *Obtain potential signatures of max length Nv’:* The total number of possible signatures ${\mathrm{N}}_{\mathrm{s}}$was defined by Equation (1). These signatures contained a different number of variables, from size 1 to size. A random selection of three signatures from size 2 to size was made. A total of *S* signatures were therefore selected (see Table S2 in the supplementary data File S2) for the comparison using Equation (2).

(1) \begin{equation*} {N}_s={2}^{N_{v^{\prime }}}-1 \end{equation*}

(2) \begin{equation*} S=3\left({N}_{v^{\prime }}-2\right) \end{equation*}

- *Define learning and validation sets:* A total of *k* = 50 random training partitions were generated from the original dataset, using a resampling rate *P* = 0.5 for downstream analysis. The function *createDataPartition* from the R-cran package caret [27] was used to create these partitions.

The detailed result of Step 1 and Step 2 on the three datasets is described in Table 2.

#### Step 3: RF models and AUCs

Each RF implementation was computed with the $\mathrm{ntree}$ parameter for a total of times based on (Equation 3); where *S* is the number of signatures selected based on Equation (2), is the number of random partitions used, and *q* is the number of models generated per partition.


(3) \begin{equation*} {\mathrm{RF}}_r=S.k.\kern0.5em q \end{equation*}

For the current study, each RF implementation was sent to an individual computational node for model training and validation, with *k* = 50 and *q* = 25. Each node was, therefore, handling computations, which resulted in models and AUCs. Metrics for each model validation were also computed with the R-package *MLmetrics* [28]. The time was measured before and after each modelization. To get the average AUC of an implementation, the AUC was averaged across runs. Similarly, the time to process the model was averaged across runs to get the average runtime of the implementation.

To measure the runtime, we selected computational nodes having the same characteristics. We used the nodes from the CECI’s Dragon1 cluster hosted by the UMons University Belgium, which provided 416 CPUs distributed on 26 nodes and 128GB of RAM. The CPUs used were SandyBridge processors of 2.60GHz.

- Step 4: Hyper-stability

For a given RF implementation and resampling–signature combination (totaling $c=S.k$ combinations), *q* = 25 AUCs were generated. The coefficient of variation (CV) of this set of AUC values was computed. This CV measures the average variability of AUCs around the mean AUC, defined by Equation (4), where $\mathrm{sd}$ represents the AUC standard deviation and $\overline{x}$ is the AUC mean. It ranges between 0 and 1.


(4) \begin{equation*} \mathrm{CV}=\mathrm{sd}\!\left/ \!\overline{x}\right. \end{equation*}

Equation (4) was calculated for each resampling-signature combination tested. At this point, a total of $c$ CV values were generated. Stability scores were calculated using all $\mathrm{CV}\le t$ values only, where $t$ represents the stability threshold. For this study, since we want to assess the hyper-stability of the RF methods, we set $t=0$. Thus, stability scores were computed based only on $\mathrm{CV}==0$ values. Two novel hyper-stability scores were created: a resampling-dependent (HRS) score and a signature-dependent (HSS) score. For HRS, the count of signatures with CV == 0 (named ${S}_0$) was averaged across the total number of signatures (named $S$) tested for a given resampling ${k}_n$ and was termed $\mathrm{HR}$, as displayed by Equation (5). The mean of the set of nonzero HR values obtained across all the $k$ resamplings selected (in this study, $k=50$) was then used to create the HRS score, which describes the hyper-stability of the RF implementation on a resampling coordinate. The HSS score, in turn, averages the number of resamplings with CV == 0 (denoted ${k}_0$) across the total number of resampling $k$ tested for a given signature ${S}_n$ and is referred to as $\mathrm{HS}$, as displayed by Equation (6). The mean of the set of non-zero HS values obtained across all the $S$ signatures tested (the total number of signatures $S$ tested varies from one dataset to another) was then used to create the HSS score, which describes the hyper-stability of the RF implementation on a signature coordinate.


(5) \begin{equation*} {\mathrm{HR}}_{k_n}={S}_0\!\left/ \!S\right. \end{equation*}

(6) \begin{equation*} {\mathrm{HS}}_{S_n}={k}_0\!\left/ \!k\right. \end{equation*}

We used R version 3.3.1 for all the analyses detailed in the current study. Table 1 listed all RF implementation R-packages and their versions used in the current study.

## Results

### Hyper-reproducibility of the AUCs

**Figure 2:**
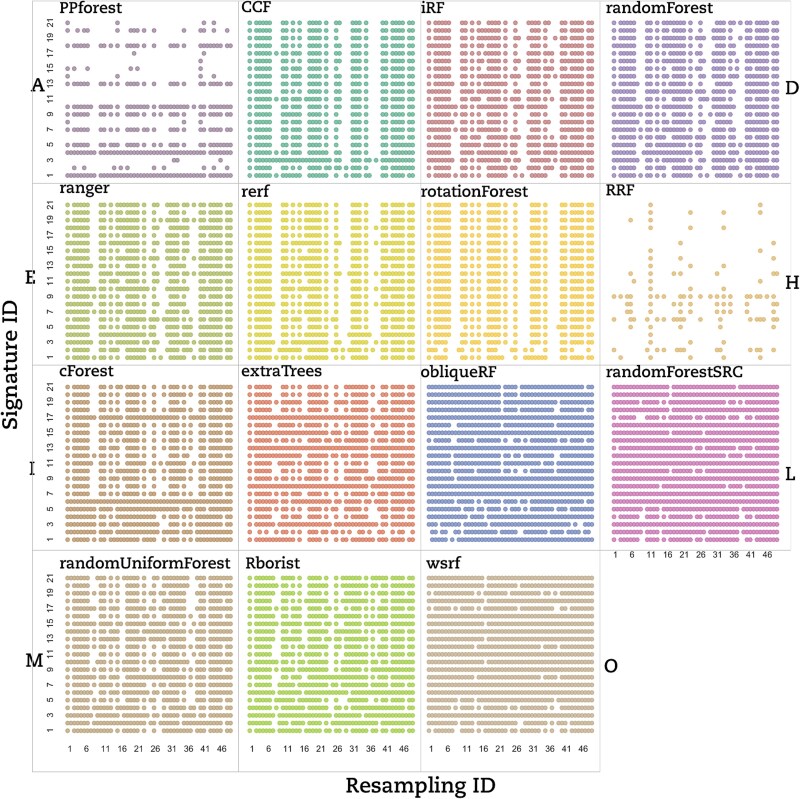
Dot matrix of AUCs’ coefficient of variation is equal to 0 for all RF implementations. Coefficient of variation equal to 0 for *q* = 25 AUCs obtained for each signature–resampling combination per RF implementation for the LUSC dataset. Each dot corresponds to 25 equal AUCs (CV == 0) for a signature-resampling combination.

To highlight the best RF strategy, the AUCs generated have to be with a high degree of precision. In this context, we used the CV to assess the AUC’s dispersion measured over 25 models generated for each signature–resampling combination. Each RF implementation was tested on a set of signature–resampling combinations selected from each of the three datasets considered in this study. We used a dot-matrix plot to visually inspect the reproducibility of AUCs for each RF implementation. A dot on the matrix means that the 25 models generated from the corresponding signature–resampling combination lead exactly to the same AUC value. In Fig. 2, a dot matrix displays all the LUSC dataset for CV == 0, obtained for all combined signature–resampling of each RF implementation.

**Figure 3:**
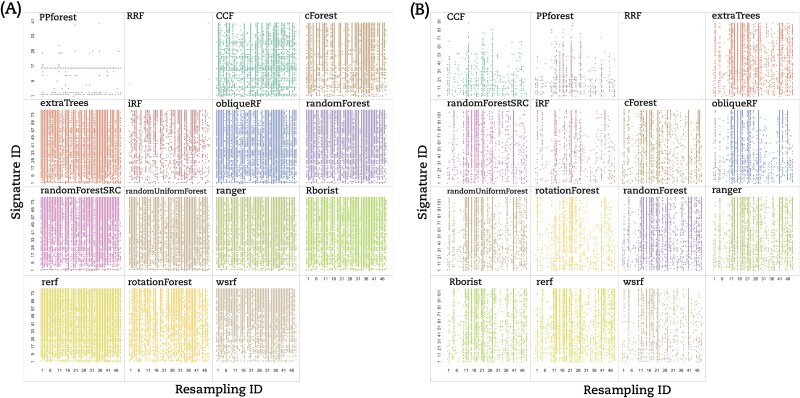
Dot matrix of AUCs with a coefficient of variation equal to 0 for all RF implementations tested. Coefficient of variation equal to 0 for *q* = 25 AUCs obtained for each signature-resampling combination per RF implementation for the (A) BRCA dataset and (B) THCA dataset. Each dot corresponds to 25 equal AUC values (CV == 0) for a signature–resampling combination.

Based on that, we classified the RF implementations into three groups based on the lack of reproducibility. (i) The signature dependent group (Type A) contained only PPForest, which displayed multiple blank rows; (ii) The resampling dependent group (Type B) encompassed CCF, Rerf, randomForest, iForest, and ranger, which presented numerous empty columns; and (iii) the signature–resampling group (Type C) was composed of the remaining implementations where no trend neither in signature nor in resampling could be found. Aside from our classification, RRF showed only a few stable signature–resampling combinations and was therefore unclassified. RF implementations switched between groups depending on the dataset under study (Fig.3A for the BRCA dataset and Fig. 3B for the THCA dataset).

### Hyper-stability scores

**Figure 4:**
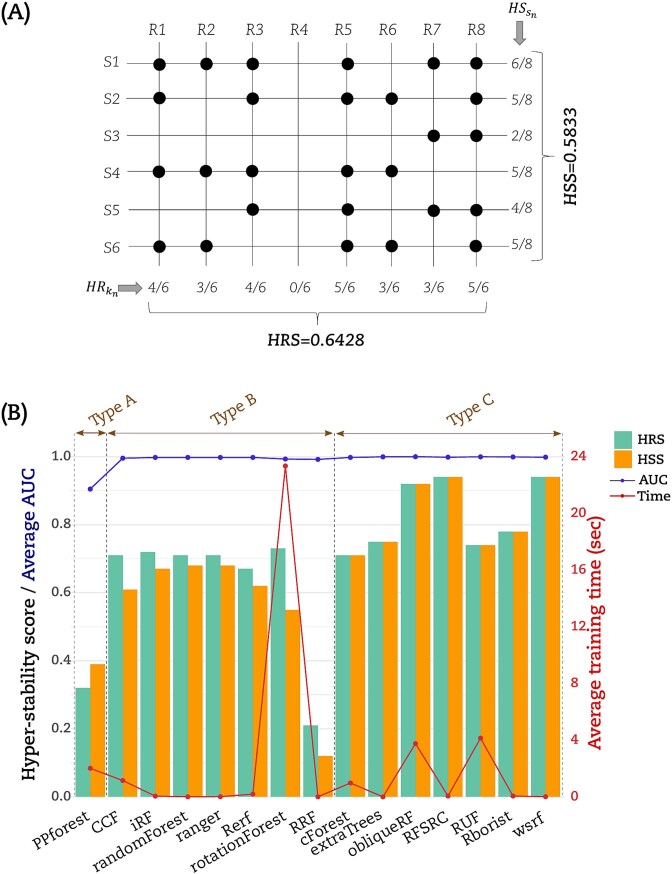
Random Forest hyper-stability scores. (A) Example of calculating the HR [Equation (5)], the HS [Equation (6)], HRS, and HSS. (B) HRS score and HSS score were displayed as bars in the plot for each RF implementation for the LUSC dataset, using the left scale of the graph. Average AUC of each RF implementation was reported with a blue line using the left scale of the graph. Average time to process a model was reported with a red line using the right scale of the graph.

**Figure 5:**
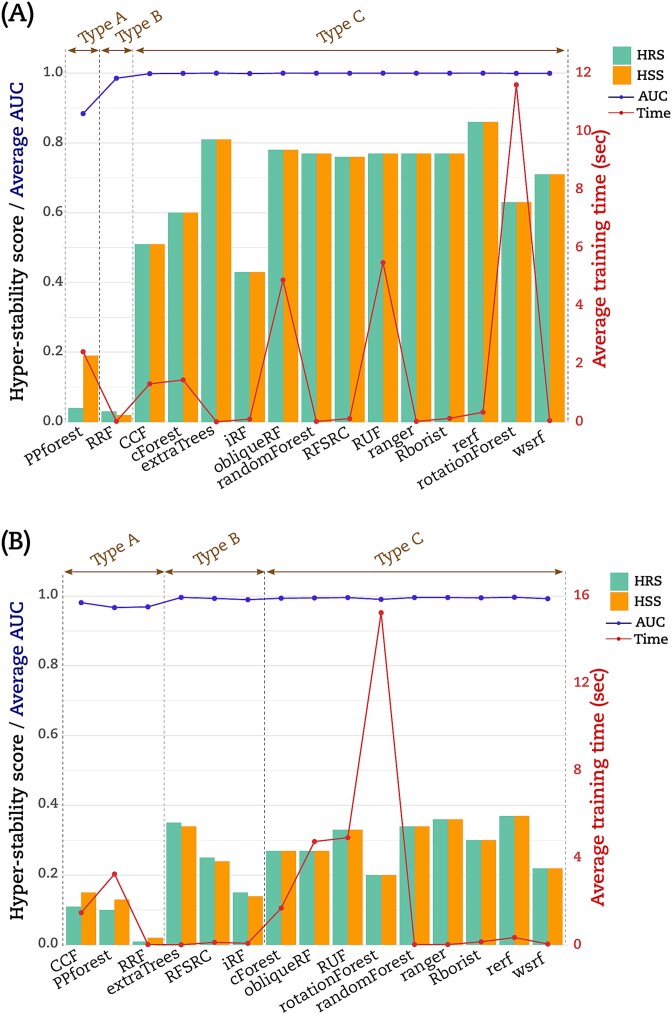
Random Forest hyper-stability scores HRS and HSS for the (A) BRCA dataset and (B) THCA dataset.

We computed the hyper-stability scores for resampling (HRS) and signature (HSS) to further compare the RF implementations according to (Equations (5) and (6)) (see Materials and Methods). The dot matrices (Figs 2 and 3) were used to calculate HRS and HSS for the three datasets. For illustration, Fig. 4A displayed an example on how to calculate HRS and HSS from the dot matrix. A bar chart to display these HRS (green bars) and HSS (orange bars) for the LUSC is given in (Fig. 4B). The Type A group PPforest method shows a combined score below 0.4. The Type B implementations show almost similar values of HRS and HSS with a combined value ≥0.65. Furthermore, among the Type C group, only obliqueRF, RFSRC, and WSRF obtained a combined score around 0.9, while the remaining implementations obtained a score below 0.8. We observed similar trends for the BRCA dataset (Fig. 5A). However, the THCA dataset displayed HRS and HSS scores below 0.4 for all implementations (Fig. 5B). More specifically, for the LUSC dataset, good scores (]0.8, 1]) were obtained for obliqueRF, RFSRC, and WSRF; moderate scores ([0.4, 0.8]) for rotationForest, CCF, cForest, extraTrees, iForest, randomForest, RUF, Rborist, ranger, Rerf, and rotationForest; and poor scores ([0, 0.4[), for RRF and PPforest. Interestingly, the poorly performing group implementations persisted across datasets (LUSC, BRCA, and THCA). The good and moderate RF implementations were inconsistent across datasets (Fig. 5A for BRCA and Fig. 5B for THCA). These results underlined the dataset dependency of the RF implementations studied here.

### Average AUC and training time

We calculated the average AUC obtained from models. We also calculated the average time to train a model. The (Fig. 4B) displayed this average AUC (blue line) and time (red line) obtained by each RF implementation for the LUSC dataset. Except for PPforest and RRF, the average AUC was equal to 1 for all the RF implementations. With an average time of 23.3 s to process a model, rotationForest was the lowest method. RF implementations CCF, cForest, obliqueRF, PPForest, and RUF processed the models with an average time between 1 and 4.1 s. The remaining RF implementations processed the models with an average time below 0.2 s. Similar trends were observed for both BRCA and THCA datasets but with a higher modelization time (Fig. 5A and B). For the THCA dataset, CCF achieved an average AUC value <1 in addition to PPforest and RRF.

### Identification of potential causes of hyper-stability score impairment

#### Algorithm taxonomic classification of RF implementations

We assessed whether the hyper-stability scores varied according to the RF implementation taxonomy previously described by Pretorius *et al*. [29]. Based on the 15 RF implementations, the following four criteria could be derived from this taxonomy: the number of layers of randomization modification; transformation or projection of the dataset; nonexhaustive search, and deterministic modifications.

Taking the randomForest original implementation as a reference, we assessed these four criteria’s impact on the HRS and HSS scores. Adding or removing layers of randomization did not improve the scores. Similarly, nonexhaustive search methods did not drastically change the scores. Indeed, extraTrees or RUF did not show the highest HRS and HSS scores. Besides, data transformation or projection might have an impact on the scores. We observed a decrease in the HRS and the HSS scores for rotationForest, PPforest, and CCF, as well as an increase in these scores for Rerf for the BRCA dataset or obliqueRF for the LUSC dataset. The deterministic modification might also impact the scores, especially for RRF, PPforest, cForest, Rerf, and WSRF. Nevertheless, no impact of this deterministic modification could be detected with the scores obtained by RUF, ramdomForest, RFSRC, Rborist, and obliqueRF. Importantly, no common impact could be linked to the Pretorius *et al*. taxonomy to the HRS and HSS scores.

#### Dataset dimension and random sampling

To assess whether the dataset’s size may impair the hyper-stability scores, we further explored the variable-sample ratio (number of variables/number of samples) in the datasets. With a resampling rate of 0.5, a total of 0.5 * 96 = 48 samples were randomly selected in each LUSC resampling, leading to a variable–sample ratio = $9\!\left/ \!48\right.=0.19$. By increasing the data resampling rate to 0.9 (hence, decreasing the data perturbation), the variable–sample ratio decreased to 0.10, which resulted in higher HRS and HSS scores for all implementations (data not shown). Despite the same number of samples in the THCA dataset, this ratio reached 0.77 for a resampling rate of 0.5, leading to HRS and HSS scores around 0.4 for all the implementations. Again, the scores could be increased over 0.8 by switching to a variable–sample ratio of 0.44 at a resampling rate of 0.9. Besides, with 28 variables and 182 samples, the BRCA variable–sample ratio reached 0.3 for a resampling rate of 0.5. Consequently, HRS and HSS scores were likely to depend on the variable–sample ratio, where good scores (>0.8) were obtained with a low variable–sample ratio (<0.5).

To evaluate whether the random resampling could impair an RF implementation’s hyper-stability, we quantified the resampling with few CV == 0. Using the dot matrix based on LUSC (Fig. 2), we observed that resampling 37 and 38 were struggling to stabilize the AUC for most RF implementations. Such a lack of stability on the LUSC dataset was particularly significant for the CCF, PPforest, Rerf, rotationForest, and RRF implementations. We made similar observations for BRCA’s resampling 6 and 34 and THCA’s resampling 6 to 10. Consequently, the presence of these problematic resamplings might contribute to the decrease of the hyper-stability scores.

#### Data connectivity

To better understand the HRS and HSS variability between the datasets, we looked into gene connectivity. Differences in connectivity might explain the performance of machine learning models. Using Weighted Gene Co-expression Network Analysis (WGCNA) [30] module preservation analysis on the 7031 genes in common between the tumor samples of the three filtered datasets, we seek for modules with highly correlated genes conserved between the three datasets. Thus, we compared BRCA > > LUSC, BRCA > > THCA, and LUSC > > THCA (Fig. S1 in the supplementary data File S1). We found that 15 modules among 25 showed low preservation for BRCA > > LUSC. When performing a module preservation analysis, genes with a similar function tend to cluster together according to a phenotype or a disease [31]. These modules were located between no preservation (blue line, Zsummary = 2) and very weak preservation (green line Zsummary = 10) region. These modules also had the lowest median rank statistic, meaning their observed preservation statistics tend to be the lowest among the other modules. Moreover, 14 highly connected genes within the grey60 module of BRCA lost their connectivity within the LUSC network (Fig. S2 in the supplementary data File S1). Similarly, for the BRCA > > THCA case, we observed that 15 modules among 25 were lowly preserved in the THCA samples, and 20 modules out of 24 showed weak preservation for the LUSC > > THCA case. These results underlined that the functional connectivity was not conserved between the three datasets and further contributed to explaining differences in HRS/HSS scores.

## Discussion

In this study, we tested the stability of 15 different RF implementations over three distinct cancer datasets. These datasets were perfectly balanced and composed of paired tumor–healthy samples. We compared RF implementations for AUC hyper-stability scores and runtime, and investigated drivers of (in)-stability.

We assessed RF’s hyper-stability-based AUC-derived HRS and HSS scores for 15 RF implementations. The AUC has become a common measure to determine the accuracy of classification models. Nevertheless, it only evaluates how much the classification model can discriminate between the classes. The AUC could, therefore, be misleading and might suffer from the following drawbacks [32]: (i) AUC ignores the probability values of the samples; (ii) it includes less interesting regions on the ROC plot; (iii) it does not reflect the intended use of the model; and (iv) it does not provide information about the spatial distribution of model errors. Besides, the AUC might be insensitive to strongly associated disease features added to the model [33]. Moreover, AUC displays a high dispersion, especially for imbalanced or small sample sets [34]. AUC alternatives can provide useful measures of performance for prognostic models [34]. Examples are the Pietra index and the standardized Brier or scaled Brier scores. These alternatives should be considered for future calculation of the hyper-stability scores.

In this work, we built on AUC for different scenarios of resampling and signature combinations to derive hyper-stability scores HRS and HSS. The proposed methodology tried to assess RF inherent randomness while keeping the external randomness under control. While we measured the exact same AUC from 25 models, our system was not deterministic, as defined by Padhye *et al*. [35]. Indeed, rule extraction showed that the genes, the thresholds, and the number of steps used could differ in each RF model (Tables S3 and S4 in the supplementary data File S1). Consequently, our system kept the intrinsic randomness of the RF implementations and could not be considered deterministic.

Good HRS and HSS scores were obtained in our study, except for the THCA dataset at a resampling rate of 0.5. With a perfect balance between tumor and healthy samples, the small sample size might explain THCA’s low performance. Nevertheless, the LUSC dataset displayed good hyper-stability scores with the same number of samples. Specific characteristics of the RF algorithm and the nature of the application data are the main drivers of model performance (Fig. S4 in the supplementary data File S1), which we discuss next.

### Algorithm characteristics

RF algorithms may differ from Breiman’s original implementation in their randomization and deterministic components. Except for the tree selection and the ensemble compilation, the 15 chosen RF implementations covered all the taxonomy criteria listed by [36]. The following characteristics could therefore impact AUC and therefore the hyper-stability performances:

(1) The number of trees; A model with more trees is better [37]. Our results were based on 500 trees for each RF implementation. Indeed, except for RRF, the resulting OOBerr stabilized for most RF implementations after 500 trees (Fig. S3C in the supplementary data File S1). RRF struggled to stabilize the OOBerr after 500 trees, which might explain its poor hyper-stability over all the datasets.
(2) The sources of randomization encompass selecting samples, sampling the features, and selecting the splitting point [36]. The randomization component deals with the insertion or deletion of randomization layers and the modification of the random sampling procedure.
(3) The deterministic modifications, which encompass oblique or orthogonal splits, impurity measure, and penalization [36]. The deterministic component deals with the tree construction, data transformation, type or rules to split, impurity measure, or variable penalties.

No relationships were found between the hyper-stability scores, the sources of randomization, and the deterministic modifications. However, good, moderate, and poor groups could be derived from the hyper-stability scores. Interestingly, only the poor group contained dataset-independent RF implementations, RRF, and PPForest.

For the purpose of the current study, we used an RF-based FS coupled with the Kuncheva and Spearman indices to check the overlap and the correlation of the variable ranking. The aim was to get the minimal set of important and stable variables to improve the reliability of the selection before performing the comparison. The FS used did not favor any RF implementation method; it may negatively impact the poor group. However, FS methods that promote predictivity, sparsity, and reliability are recommended and should be considered in future development such as Stabl, recently proposed in [38]. For the poor group, RRF uses regularized selected variables during FS. Further work is thus needed to study how such regularization may affect the FS used here. This work might be done for RRF and PPforest using the Linear Discriminant Analysis (LDA)-based projection pursuit index to identify projections that separate classes [39].

Conversely, the good and the moderate groups contained nonfixed RF implementations regarding the dataset studied. Indeed, each RF implementation resulted in different HRS and HSS scores when facing another dataset and thus might be classified as good or moderate.

### Dataset characteristics

Although the RF versatility between Good and Moderate groups could not be ignored, it might be linked to the training set. Indeed, the selected samples and variables should provide enough information to the model to fully recognize the patterns. A low variable–sample ratio could be critical to produce models with a high average AUC [40] and good hyper-stability. The following dataset characteristics could, therefore, impact the AUC and therefore, the hyper-stability performances:

(1) The balance between the categories: While the three datasets used in the current study were perfectly balanced between the tumor and the healthy classes, extremely imbalanced classes could harm RF behaviors [41]. To apply our methodology to nonbalanced datasets, we recommend the use of balanced or weighted RF implementations. Chen *et al*. proposed balanced and weighted RF, where balanced RF will force to deal with equally sized classes, and the weighted RF was based on cost-sensitive learning. For such skewed datasets, the precision–recall curve (PR curve) and the weighted AUC should be preferred over the ROC curve and the AUC [42, 43].
(2) The size of the training set: The number and the heterogeneity of the samples could be an essential source of instability during the biomarker selection, leading the training set to be more or less attractive for the RF [44, 45]. Subsequently, thousands of samples were recommended to reach good Kuncheva and Spearman scores [40]. However, our results achieved acceptable scores with the FS and only 192, 96, and 98 samples, meaning that a good *a priori* on the samples could circumvent this issue. Nevertheless, few resamplings displayed a low number of CV == 0 across RF implementations but impacted them similarly. A good *a priori* on the sample classes could, therefore, lead to heterogeneous random resamplings. Thus, such heterogeneity could impact the FS and the hyper-stability scores [26]. Interestingly, the variable–sample ratio appeared to be more related to the variability observed for the hyper-stability scores. By keeping the variable-sample ratio below 0.5, we obtained good hyper-stability scores (>0.8) for the implementations. For example, we observed that the AUC reproducibility decreased along with the signature size for the THCA dataset, which was the only dataset that displayed a high variable–sample ratio and an average of low hyper-stability scores. Also, the length of a signature might impact the *mtry* RF parameter and might impair their hyper-stability [46]. However, by increasing the resampling rate (hence, decreasing the data perturbation) of the training step to 0.9, the RF implementations reached good hyper-stability scores while keeping the variable–sample ratio below 0.5. Therefore, reducing the or the data perturbation for the THCA dataset could keep the variable–sample ratio below 0.5, leading to higher hyper-stability scores. Remarkably, we repeated all the modelizations on three different sets of increasing signature sizes for each dataset, and the same hyper-stability scores (average SD = 0.01) were obtained each time for each RF implementation. Subsequently, the HSS and HRS scores were more tied to the models and their over-fitting when the variable–sample ratio was kept under 0.5.
(3) The feature connectivity: By grouping the genes into modules of highly co-expressed genes, we could assess the FS bias occurring when highly connected genes are selected. Our results demonstrated the difference between the three datasets in the gene–gene connectivity across the tumor samples, meaning that the functional information was different between the datasets. While 56% of the modules displayed a low to no preservation from BRCA to LUSC (14/25) or from BRCA to THCA (14/25), 83% of the modules displayed a weak to no preservation from LUSC to THCA (20/24). Such a difference might explain the variation also observed in AUC hyper-stability between the datasets. Nevertheless, more work is needed to find a causative link between such connectivity and either the AUC hyper-stability or the data perturbation by performing module preservation between the training partitions selected.
(4) The correlations between variables within signatures: Datasets with many correlated variables may create misleading feature rankings [47]. Very few strong correlations were found within BRCA signatures (Table 2), while this dataset displayed excellent hyper-stability scores. Subsequently, the correlations between variables within the signatures could not explain the lower hyper-stability scores observed for both the LUSC and the THCA datasets.
(5) The variability of the dataset: With high entropy, datasets tend to be more sensitive to perturbation, which results in different AUC performance. This is often the case with small datasets like biological data. In this study, the FS used allowed us to maximize the class separability and separate the samples according to the tumor or healthy groups. However, while a perfect separation was observed for the BRCA and LUSC datasets, it was close to perfect for the THCA dataset. Indeed, all but four THCA samples were linked to their respective class. These few crossing-class samples might explain both the high number of THCA variables after the FS and the low AUC hyper-stabilities. Further work is therefore needed to assess if the class separability could impair the AUC hyper-stability.

In addition to the dataset and algorithm characteristics, the stability threshold selected can significantly influence stability performance, which we will discuss in the next section.

### Hyper-stability and stability threshold

All hyper-stability measurements were based on CV == 0 values, indicating that no variability in the AUC values obtained was tolerated. This strictness was essential as the three chosen datasets proved to be optimal. The RF methods effectively differentiated between healthy samples and tumors. As a result, nearly perfect AUC values were reached across several iterations of each RF implementation with various resampling–signature combinations. In this section, we illustrated how to generalize stability scores to different contexts and alternatively defined relative stability. As outlined in the Materials and Methods section, the stability threshold $t$, which controls the dispersion of AUCs, can be assigned a value ($0<t\le 1$). In such situations, the AUC values obtained were not necessarily identical. Consequently, the dispersion around the mean AUC became >0 (instead of $t=0$ selected for the hyper-stability), and we referred to this as relative stability. HRS and HSS scores are redefined as RRS (Relative-stability Resampling Sensitive) and RSS (Relative-stability Signature Sensitive), respectively. This indicated that hyper-stability represented a particular case of relative stability when *t* equals 0. The values of hyper-stability scores HRS and HSS, along with the relative stability scores RRS and RSS, are shown in Fig. S6 of the supplementary data File S1 for the THCA dataset (with a resampling rate *P* = .3). For $t=0$ (i.e. CV == 0), the dot matrices generated were either sparse or nearly empty for most RF implementations (Fig. S6.A in the supplementary data File S1). As a result, the HRS/HSS scores were consequently low (Fig. S6.B in the supplementary data File S1). Setting the stability threshold *t* = 0.002 (i.e. CV ≤ 0.002) produced denser dot matrices (Fig. S6.C in the supplementary data File S1) and led to higher stability scores (Fig. 6.D in the supplementary data File S1).

Additionally, we aimed to illustrate an instance where stability scores were derived from AUC values that are not close to perfect (i.e. <1). To achieve this, the pipeline was utilized on the dataset referenced in [6], which was used to develop a circulating miRNA-based screening signature for breast cancer. The dataset (referred here as miRNA-BRCA) included normalized expression values for 188 miRNAs from 282 healthy tumor samples, which consisted of 149 patients with treatment-naive primary breast cancer and 133 age-matched cancer-free women. The authors have designed 128 screening signatures to perform optimally on an independent dataset. From this collection, we selected *S =* 30 signatures of different lengths (from 4 to 23). Additionally, we used the following parameters to execute the pipeline: *k* = 30 (indicating the number of random partitions with *P* = .5), q = 25 models, and ntree = 500. The relative stability scores RRS and RSS were then calculated using two thresholds *t =* 0.004 and *t =* 0.008, and the results were displayed in Fig. S7 in the supplementary data File S1; results were shown for only 12 RF methods. As expected, the average AUC performance of the evaluated methods ranged between 0.70 and 0.80, with cForest attaining the highest mean AUC and PPforest the lowest (Fig. S7.B and S7.D in the supplementary data File S1). Regarding the stability of the RF methods, the dot matrices for a threshold of *t* = 0.004 were nearly full for cForest and rotationForest, while it was nearly empty for PPforest and about half full for the other methods (Fig. S7.A in the supplementary data File S1). This was reflected in the stability scores obtained. The method cForest achieved highest stability scores RRS = RSS = 0.81, followed by rotationForest RRS = RSS = 0.68. The other methods yielded stability scores below 0.35, with a lowest score for PPforest RRS = RSS = 0.03 (Fig. S7.B in the supplementary data File S1). Switching to a threshold of *t* = 0.008 resulted in significantly more populated dot matrices for most RF methods (Fig. S7.C in the supplementary data File S1), thereby improving stability scores of the RF methods (Fig. S7.D in the supplementary data File S1). These results indicated that stability scores were highly influenced by the selected threshold $t$, rather than the mean AUC performance of the methods. Furthermore, the RF methods switched among groups A, B, and C across different datasets and depended on the stability threshold $t$ selected within the same dataset.

According to the current study, the HR score intuitively depends on the robustness of the tested signature. This allows us to assess the reproducibility of AUC performance across various resamplings. In contrast, the HS score may pertain to the signature multiplicity mentioned in [48], which enables evaluation of the performance of several alternative signatures within a single resampling partition.

## Conclusion

In the current study, we demonstrated the importance of measuring RF AUC hyper-stability in the context of short BSD. We reinforced the message that no RF implementation should be used blindly for classification and on any datasets. Instead, each should be tested for its AUC performance and AUC-derived hyper-stability before the analysis. While the AUC-derived hyper-stability could reveal the dataset dependency of an RF implementation, it could also identify the origin of such reliance, telling whether an RF implementation is signature or resampling dependent. Therefore, the hyper-stability scores measured a trustable difference that should be taken into account while comparing the RF implementations. Moreover, the modelization time could further help discriminate RF implementations with equal hyper-stabilities. Consequently, the AUC hyper-stability and the modelization time reinforce the average AUC message and guide the researchers towards the best RF strategy for short biomarker signature discovery or other fields.

**Key Points**

- We introduced a new metric, the AUC hyper-stability, to be used in complementary with the average AUC to better measure the performance of a Machine Learning (ML) model in the context of short biomarker signature discovery.
- For illustration, we conducted a performance comparison of 15 RF implementations applied to three datasets from TCGA database. The performance metrics included AUC, AUC hyper-stability, and runtime.
- The AUC hyper-stability metric is able to discriminate RF implementations that show similar AUC performances.
- This new metric can therefore help researchers in the choice of the best ML method to get stable short predictive signatures. The choice can be done by taking a tradeoff between the average AUC performance, the hyper-stability scores, and the modeling time.

## Supplementary Material

File_S1_updated_bbaf318

File_S2_bbaf318

## Data Availability

The pipeline used in the current study is made of two components; each one was implemented and versioned separately and then having its own package. The first component concerns the feature selection and was implemented via the R package stabFS, and can be found in the Gitlab repository at https://gitlab.com/a.debit/stabfs, and available in the Figshare platform at https://doi.org/10.6084/m9.figshare.24878646.v1. The second component deals with the comparison and was implemented in the R package called compareRf. CompareRf is publicly accessible at https://gitlab.uliege.be/giga-humangenetics/tools/rpackages/comparerf. The TCGA datasets used in this research can be downloaded from https://www.cancer.gov/tcga. By providing open access to the data and source code, we aim to ensure transparency, reproducibility, and ease of collaboration within the research community.
