# Supplementary material for: Assessing Random Forest self-reproducibility for optimal short biomarker signature discovery
Source: Brief Bioinform. 2025 Jul 11;26(4):bbaf318. doi: 10.1093/bib/bbaf318 (PMC12245662; doi:10.1093/bib/bbaf318)
Supplement: File_S1_updated_bbaf318 [file file_s1_updated_bbaf318.pdf]

Supplementary data: Assessing Random Forest self-reproducibility for optimal short biomarker signature discovery

Supplementary Methods

Stable RF-based Feature Selection

A total of $k = 50$ balanced partitions were randomly defined from the original TCGA datasets, using a resampling rate $p = 0.9$. For each random partition, $q = 25$ RF model importances were calculated using the *randomForest* R-package with the default parameters [1]. The Mean Decrease in Accuracy MDA and the Mean Decrease in Gini MDG were computed for all the variables over the q models. The MDA and MDG were respectively ranked, and their average rank was used to rank the variables for each partition. The k ranking sequences obtained were used to assess the stability of the first 200 variables using the Spearman (the correlation aspect) and the Kuncheva (the overlap aspect) statistics from the R-package *OmicsMarkeR.* The first local maxima, common between Spearman and Kuncheva, gave the minimal set of important variables Nv'. Good stability values i were obtained for the Kuncheva index ($i \geq 0.8$), which means the models selected almost the same variables at each run [2].

Assessing RF randomness within identical AUCs

To assess whether the RF implementations keep their intrinsic randomness, we extracted the rules of two distinct models that produced the same AUC. The R package *inTrees* was used to extract these rules [3]. Table S3 displays an example of the rules from two randomly selected randomForest models, trained on one signature-resampling combination. A more detailed example on each dataset is also available in Table S4, which displays the rules from two random randomForest models, trained on three signature-resampling combinations. The genes, the thresholds, and the number of steps used were different in the three rules and the two models, while the AUC was precisely = 1. These results indicated that the inherent randomness was conserved in our methodology.

Supplementary Results

Feature Selection and number of trees

To keep the minimal amount of important variables at $Nv' < 50$ for the purpose of the short BSD, we computed the Kuncheva and Spearman indices from the variance-filtered TCGA datasets. On the top 200 most important variables, the cardinality corresponding to

the first local maxima of Kuncheva and Spearman indices were matched to the rank distribution of each variable (Figure S3A and S3B, and *Nυ'* in Table 2). Finally, we assessed the RF parameter *ntree* to use in the modelizations. We set it as the common number of trees that reached a minimal and stable OOBerr across RF methods, and N_t=500 was obtained (Figure S3C). The parameters selected for this step were summarized in (Table 2).

Efficiency of the feature selection used

To assess whether the features selected with the FS led to the separation of the tumor and healthy classes, we applied a PCA on each dataset. Using the *plotPCA* function of the EDASeq package [4], the (Figure S5) displays those PCAs before and after applying the FS. For BRCA (Figure S5A), 89% of the variability of the dataset was explained by the two first components after the FS, while only 40% was explained on the original dataset. For all three datasets, the FS increased the variability captured by of the variability was explianed by PC1 and PC2 in the original (raw) dataset. The same trend was observed for LUSC and THCA datasets. Nevertheless, four samples of the THCA dataset were not well clustered after the FS. Consequently, except for a few resistant THCA samples, the features selected could separate the samples according to their expected category.

Correlation between the variables within a signature

To understand how the combinations of the selected features may bias the modelizations, we calculated the correlation between each signature's variables. Therefore, we looked at the overall correlations observed between the variables and the percentage of highly-correlated variables within each signature (see Table 2). We calculated the overall correlation as the average Pearson correlation between the variables selected by the FS in the whole dataset. We set the percentage of highly-correlated variables within a signature as the proportion of signatures for which the variables achieved the Pearson correlation ≥ 0.75 over all the resamplings. The LUSC dataset displayed strong variable correlations within each signature and for most of the resamplings. On the opposite, only a few strong correlations were observed for the BRCA and THCA datasets. Interestingly, these strong correlations stuck to few resamplings. By resetting the signature set with new signature combinations, similar patterns of correlation were observed.

Supplementary Discussion

The FS developed in the current methodology was mostly based on a previous observation made by Alelyani et al. [5]. This FS aimed at capturing the most stable variables among thousands from each dataset.

Gene expression datasets with small-size samples and high dimensional features were previously described as suffering from intrinsic instability [2,5,6]. Interestingly, we did not observe such an effect on the three datasets, maybe due to the prior filtering based on paired tumor / healthy samples.

The Kuncheva (overlap) and Spearman (correlation) indices were used since we expected high ranking correlations with such a number of variables. The Spearman index was chosen over the Pearson index because of its compatibility with feature weighting [6,7]. The minimal number of stable features was set at the first local maxima reached by both indices as proposed by Alelyani et al. [5] and refined by discarding variables with a large dispersion of their ranking (Figure S3B). Such visual selection could influence the number of variables obtained, and subsequently, this step should be improved for a more objective choice.

The FS used the variable importance measures MDG and MDA of the original RF algorithm described by Breiman in 2001 [8] as a standard. Whereas the randomForest package was used for the FS, our results did not rank it as the best algorithm. With 10 of the 15 RF methods implementing an FS algorithm we expected up to ten different selected features to start our comparisons, which would have been hard to handle. Nevertheless, this study would benefit from results obtained based on the nine other FS. Besides, the FS used a data perturbation of 0.9 together with an average of 50 rounds of FS. While other perturbations were possible, we aimed at reaching a set with the most stable variables for each dataset with an average AUC close to 1. Such a high average AUC was mandatory to demonstrate the differences in AUC hyper-stabilities for the purpose of the current study.

References

1. Liaw, A.; Wiener, M. Classification and Regression by randomForest. 2002, 2.
2. Wang, H.; Yang, F.; Luo, Z. An Experimental Study of the Intrinsic Stability of Random Forest Variable Importance Measures. BMC Bioinformatics 2016, 17, 60, doi:10.1186/s12859-016-0900-5.
3. Deng, H. Interpreting Tree Ensembles with inTrees. Int J Data Sci Anal 2019, 7, 277–287, doi:10.1007/s41060-018-0144-8.
4. Risso, D.; Schwartz, K.; Sherlock, G.; Dudoit, S. GC-Content Normalization for RNA-Seq Data. BMC Bioinformatics 2011, 12, 480, doi:10.1186/1471-2105-12-480.

5. Alelyani, S.; Liu, H.; Wang, L. The Effect of the Characteristics of the Dataset on the Selection Stability. In Proceedings of the 2011 IEEE 23rd International Conference on Tools with Artificial Intelligence; November 2011; pp. 970–977.
6. He, Z.; Yu, W. Stable Feature Selection for Biomarker Discovery. Comput Biol Chem 2010, 34, 215–225, doi:10.1016/j.compbiolchem.2010.07.002.
7. Saha, S.K.; Sarkar, S.; Mitra, P. Feature Selection Techniques for Maximum Entropy Based Biomedical Named Entity Recognition. Journal of Biomedical Informatics 2009, 42, 905–911, doi:10.1016/j.jbi.2008.12.012.
8. Breiman, L. Random Forests. Machine Learning 2001, 45, 5–32, doi:10.1023/A:1010933404324.

Supplementary figures

Figure S1. Evaluation of module preservation between tumor samples of the three datasets. (A) BRCA >> LUSC; (B) BRCA >> T HCA; and (C) LUSC >> THCA. Horizontal dashed lines on the preservation Zsummary plots delimit weak preservation zone. Modules located above the blue line are not preserv-ed.

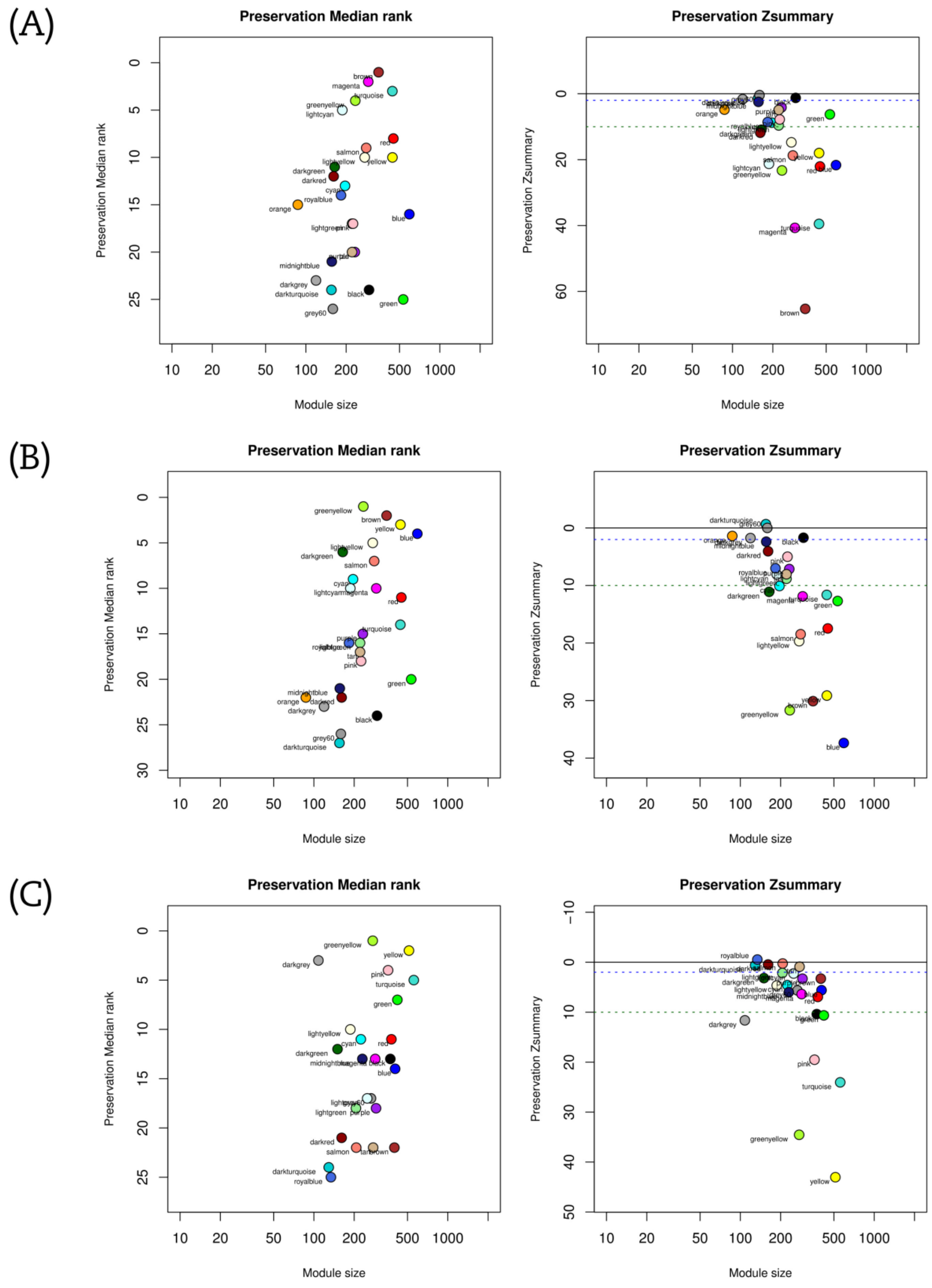

Figure S2. Example of the change in connectivity between genes within the grey60 module for the BRCA >> LUSC analysis. (A) The 14 most connected genes within the grey60 module in BRCA tumor samples are selected, and (B) the corresponding connections in the LUSC network are calculated and plotted. The network is generated using VisANT software.

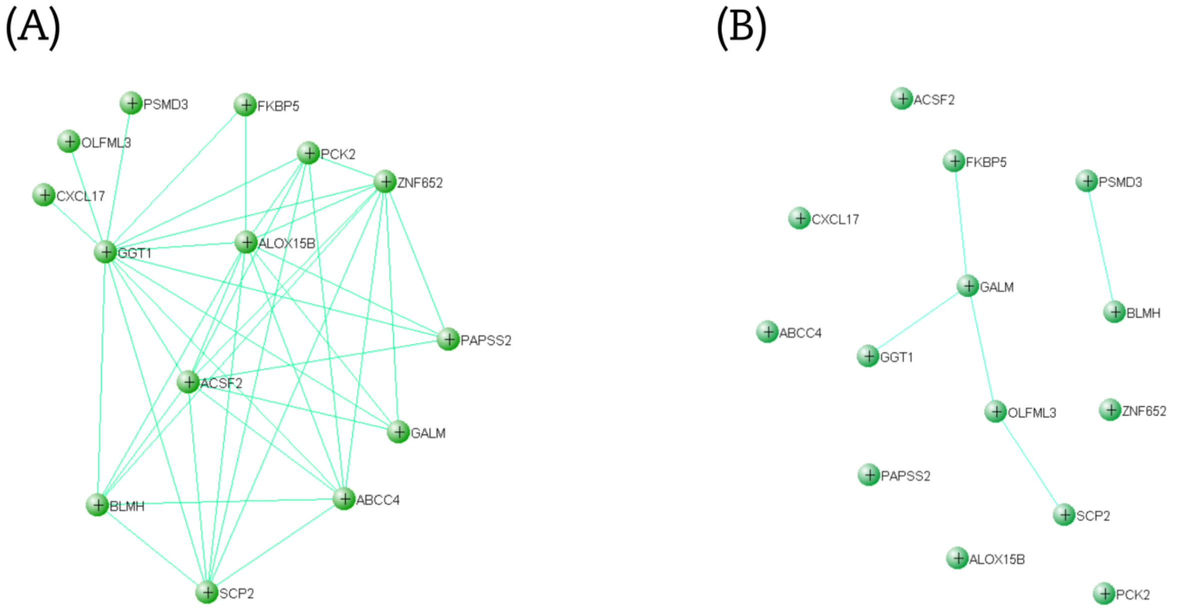

Figure S3. Feature selection on the BRCA-TCGA dataset. (A) Stability indices for the FS were calculated for an increasing cardinality from 10 to 200 with a step of 10. The minimal number of stable variables (30 for the BRCA-TCGA dataset) was set as the first local maxima observed on the Kuncheva index (vertical blue line); (B) The rank distribution of the top 200 variables. For each variable, all the 1250 ranks were displayed as a boxplot. The variables were then ordered based on their average rank. The minimal number of important variable obtained from figure a was reported as a vertical blue line. The adjustement to this number was reported here as a vertical red line; (C) The prediction error (OOB error) was calculated for the methods implementing the OOB concept. The parameters used to compute these OOB errors were based on results obtained with figure A, B. The error was stable after 500 trees (vertical blue line) for all the implementations.

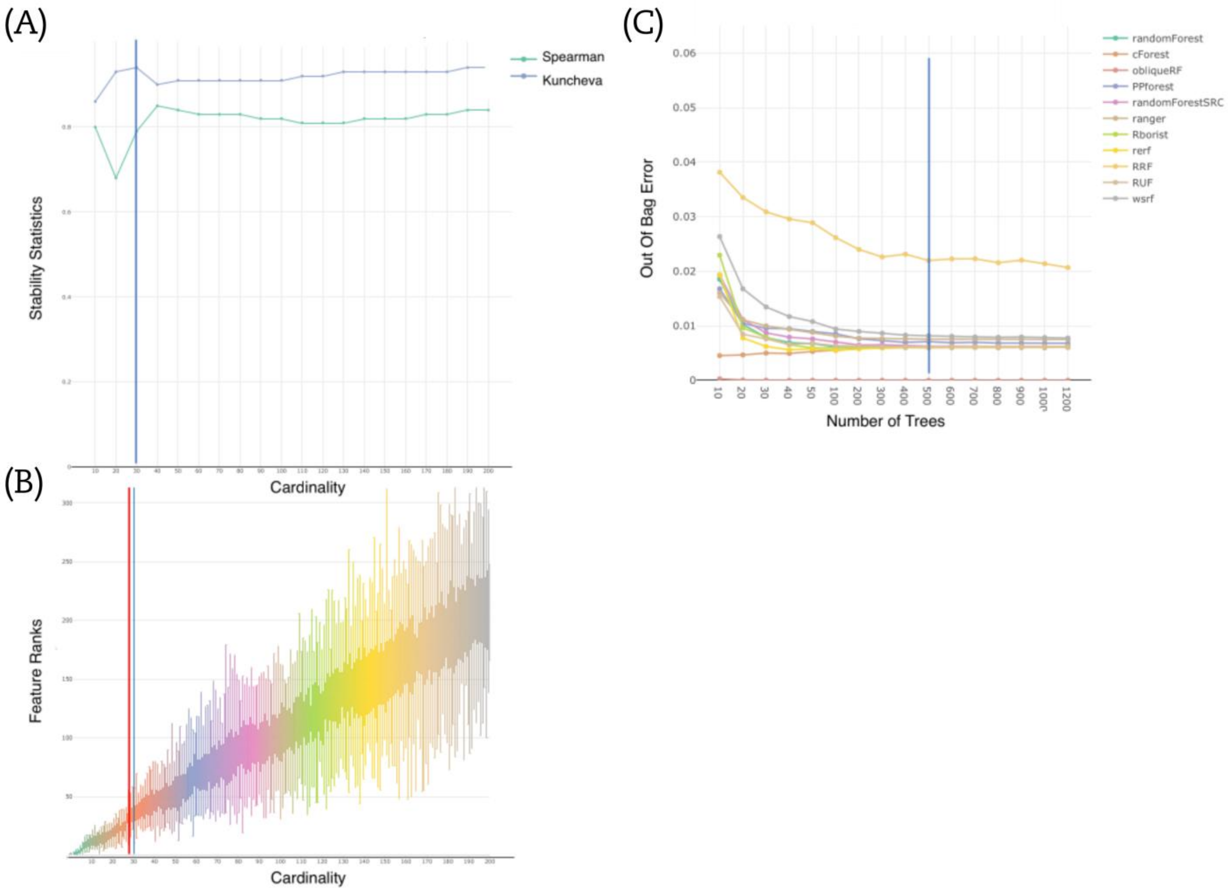

Figure S4. AUC performance impacting factors. A diagram showing factors impacting the AUC performance of an RF algorithm. Some factors concern dataset characteristics, others relate on the construction of RF algorithm. Both randomization sources and deterministic modifications components are used to construct RF variants (inspired from [37])

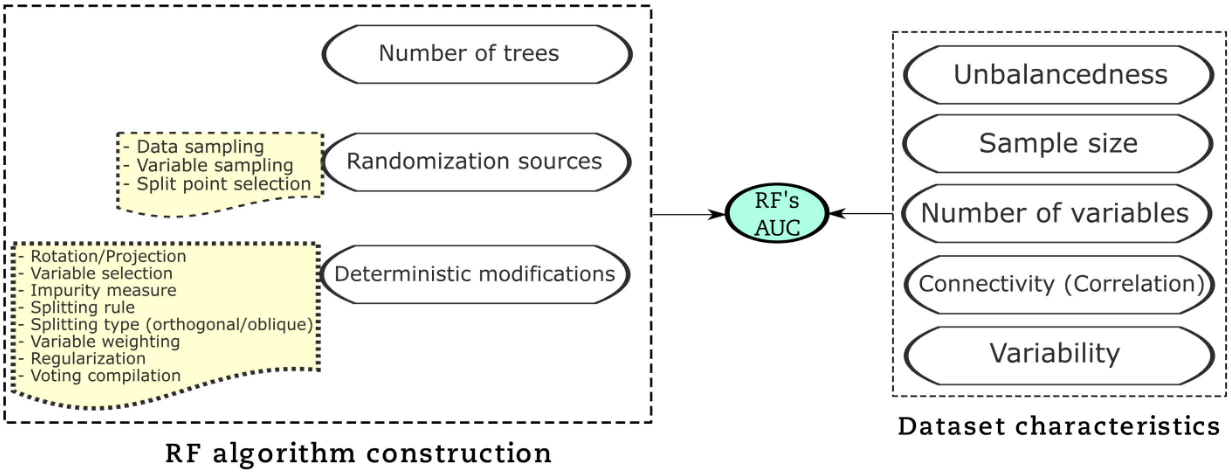

Figure S5. PCA of samples before and after the Feature Selection PCA projection of the 2 first principal components for (A) BRCA; (B) LUSC, and (C) THCA datasets. For each graph, the tumor samples are in red and their paired healthy tissues are in green. The Feature Selection increases the separatibility of the samples according to their respective class (tumor or healthy).

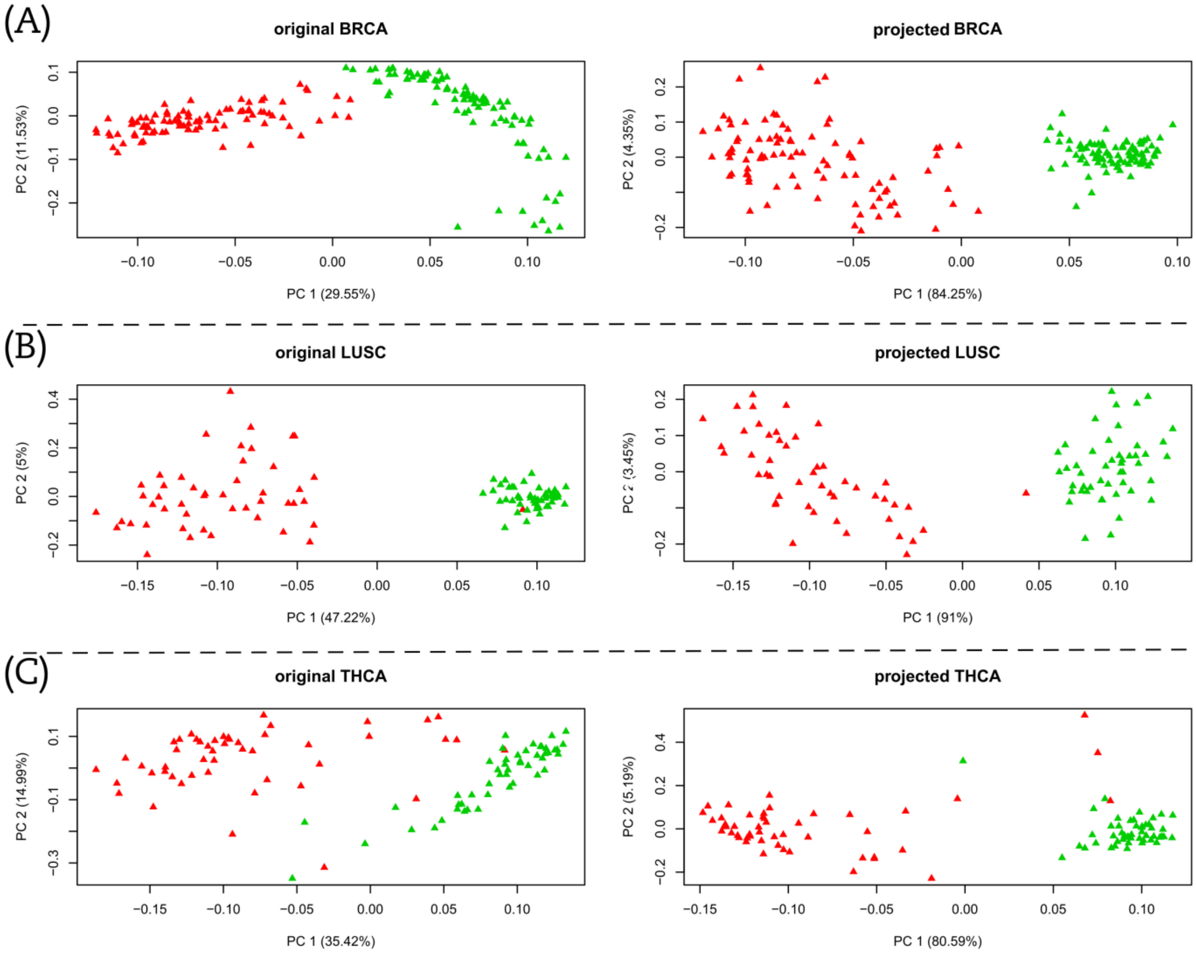

Figure S6. Stability scores for TCGA-THCA dataset for different thresholds of CV: (A) dot matrix for 14 RF methods for t=0 (i.e., CV==0), (B) Hyper-stability scores HRS/HSS, mean AUC and runtime for t=0 corresponding to the dot matrix in A, (C) dot matrix for 14 RF methods for t=0.002 (i.e., CV<=0.002), and (D) Stability scores RRS/RSS, mean AUC and runtime for t=0.002
corresponding to the dot matrix in C.

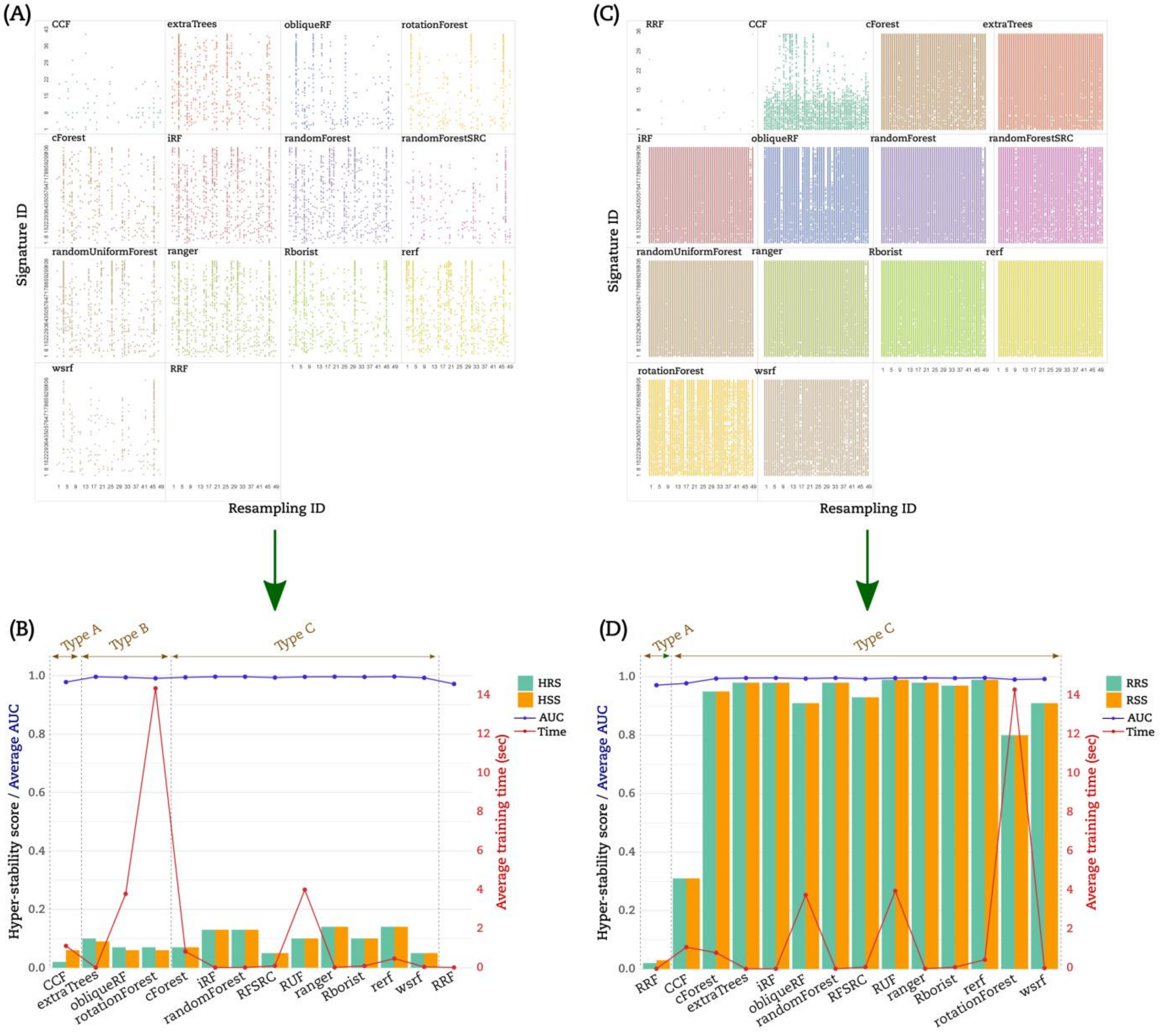

Figure S7. Stability scores for miRNA-BRCA dataset for different thresholds of CV: (A) dot matrix for 11 RF methods for t=0.004 (i.e., CV<=0.004), (B) Stability scores RRS/RSS, mean AUC and runtime for t=0.004 corresponding to the dot matrix in A, (C) dot matrix for 11 RF methods for t=0.008 (i.e., CV<=0.008), and (D) Stability scores RRS/RSS, mean AUC and runtime for t=0.008 corresponding to the dot matrix in C.

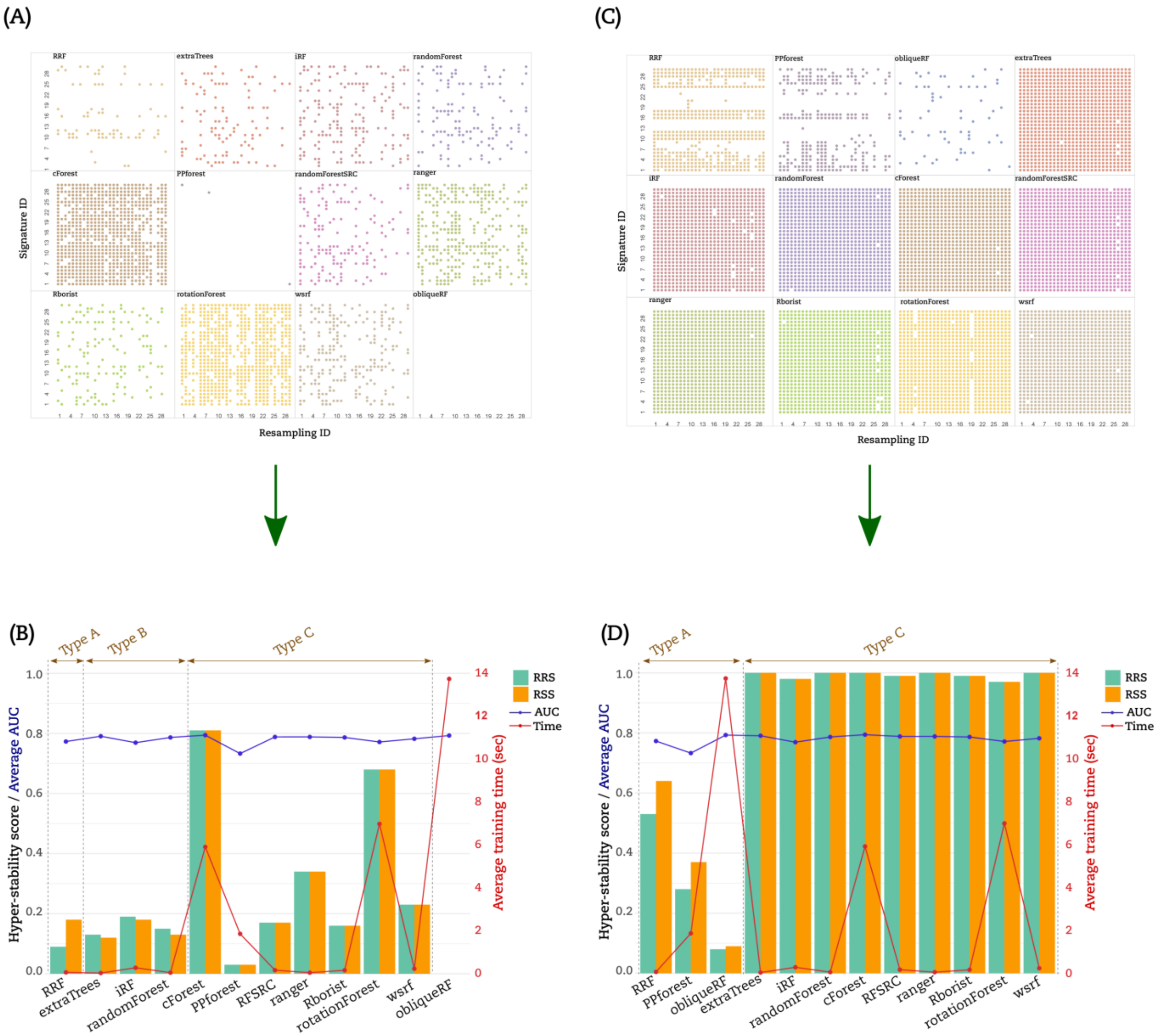

Supplementary tables

Table S1. Description of different symbols used in the formulas.

Symbol	Description
Ns	Number of all possible combinations (signatures) generated from Nv' variables
Nv	Number of variables of TCGA datasets after the normalization and filtering step
Nv'	Minimum number of variables to be kept for downstream analysis. This number is determined by FS and stability indices.
Nt	Finetuned value of $ntree$ parameter for all RF implementations.
S	Number of different sized signatures randomly selected from the set of Ns signatures
p	Resampling rate. It refers to the percentage of data that goes to training partition after a balanced random sampling from the original data.
k	Number of partitions randomly sampled from the dataset
q	Number of intrinsic RF models constructed/used for each partition-signature combination, and for each RF implementation
RFr	Total number of runs of each RF implementation
CV	Coefficient of variation of the $q = 25$ AUC values generated for each partition-signature combination, and for each RF implementation
s	Sample standard deviation
$\bar{x}$	and $\bar{x}$ Sample mean
S_0	Number of signatures with $CV == 0$ for a given resampling partition
k_0	Number of resampling partitions with $CV == 0$ for a given signature
HR_{k_n}	Hyper-stability of the resampling k_n
HS_{S_n}	Hyper-stability of the signature S_n
HRS	Mean of non-zero HRs across all resampling partitions
HSS	Mean of non-zero HSs across all signatures

Table S3. Rules randomly extracted from two randomForest models trained on the same partition and the same signature for the LUSC dataset.

rule/step	signature	resampling	model	condition	prediction
1/1	s3	r47	model1	PTPRB>2385.9889	normal
1/2	s3	r47	model1	Else	tumor
1/1	s3	r47	model2	GPIHBP1<=451.4196	tumor
1/2	s3	r47	model2	Else	normal

Table S4. Three sets of rules randomly extracted from two randomForest models. The RF models were trained on the same partition and the same signature for the BRCA, the LUSC, and the THCA datasets.

dataset	step	rule	signature	resampling	model	length	frequency	error	condition	prediction
BRCA	1	1	signature2	resampling1	model1	2	0.50	0	COL10A1<=899.8924 & SDPR>543.7528	normal
BRCA	2	1	signature2	resampling1	model1	1	0.50	0	Else	tumor
BRCA	1	1	signature2	resampling1	model2	2	0.50	0	COL10A1>84.6616 & HLF<=648.1244	tumor
BRCA	2	1	signature2	resampling1	model2	1	0.50	0	Else	normal
BRCA	1	2	signature30	resampling48	model1	2	0.50	0	EZH1>983.8384 & GPRASP1>278.3492	normal
BRCA	2	2	signature30	resampling48	model1	1	0.50	0	Else	tumor
BRCA	1	2	signature30	resampling48	model2	1	0.48	0	CALCOCO1>2232.891	normal
BRCA	2	2	signature30	resampling48	model2	1	0.51	0.02	EZH1<=1269.0316	tumor
BRCA	3	2	signature30	resampling48	model2	1	0.01	0	Else	normal
BRCA	1	3	signature70	resampling21	model1	1	0.50	0	MMP11<=406.0273	normal
BRCA	2	3	signature70	resampling21	model1	1	0.50	0	Else	tumor
BRCA	1	3	signature70	resampling21	model2	1	0.50	0	PPP1R12B<=2025.1838	tumor
BRCA	2	3	signature70	resampling21	model2	1	0.50	0	Else	normal
LUSC	1	1	signature3	resampling47	model1	1	0.50	0	PTPRB>2385.9889	normal
LUSC	2	1	signature3	resampling47	model1	1	0.50	0	Else	tumor
LUSC	1	1	signature3	resampling47	model2	1	0.50	0	GPIHBP1<=451.4196	tumor
LUSC	2	1	signature3	resampling47	model2	1	0.50	0	Else	normal
LUSC	1	2	signature11	resampling27	model1	1	0.50	0	TGFBR2>7580.5877	normal
LUSC	2	2	signature11	resampling27	model1	1	0.50	0	Else	tumor
LUSC	1	2	signature11	resampling27	model2	1	0.50	0	GPIHBP1>451.4196	normal
LUSC	2	2	signature11	resampling27	model2	1	0.50	0	Else	tumor
LUSC	1	3	signature21	resampling3	model1	1	0.50	0	GPR116<=7183.4553	tumor
LUSC	2	3	signature21	resampling3	model1	1	0.50	0	Else	normal
LUSC	1	3	signature21	resampling3	model2	1	0.50	0	ESAM>2673.1366	normal
LUSC	2	3	signature21	resampling3	model2	1	0.50	0	Else	tumor
THCA	1	1	signature14	resampling5	model1	2	0.50	0	DLG4>381.1242 & EPHB1<=570.4257	tumor
THCA	2	1	signature14	resampling5	model1	1	0.50	0	Else	normal
THCA	1	1	signature14	resampling5	model2	2	0.50	0	AGPAT4>93.7298 & C6orf168<=99.0904	normal
THCA	2	1	signature14	resampling5	model2	1	0.50	0	Else	tumor
THCA	1	2	signature65	resampling23	model1	2	0.50	0	GALE<=498.265 & ODZ1>19.2609	normal
THCA	2	2	signature65	resampling23	model1	1	0.50	0	Else	tumor
THCA	1	2	signature65	resampling23	model2	2	0.50	0	METTL7B<=291.4223 & ODZ1>19.2609	normal
THCA	2	2	signature65	resampling23	model2	1	0.50	0	Else	tumor
THCA	1	3	signature101	resampling42	model1	2	0.50	0	FHOD1<=876.5515 & SRCIN1<=210.8955	normal
THCA	2	3	signature101	resampling42	model1	1	0.50	0	Else	tumor
THCA	1	3	signature101	resampling42	model2	2	0.50	0	MCTP2>340.0583 & METTL7B<=289.4312	normal
THCA	2	3	signature101	resampling42	model2	1	0.50	0	Else	tumor
